# Diagnostic performance of [^68^Ga]DOTATATE PET/CT, [^18^F]FDG PET/CT, MRI of the spine, and whole-body diagnostic CT and MRI in the detection of spinal bone metastases associated with pheochromocytoma and paraganglioma

**DOI:** 10.1007/s00330-024-10652-4

**Published:** 2024-04-16

**Authors:** Abhishek Jha, Mayank Patel, Alexander Ling, Ritu Shah, Clara C. Chen, Corina Millo, Matthew A. Nazari, Ninet Sinaii, Kailah Charles, Mickey J. M. Kuo, Tamara Prodanov, Babak Saboury, Sara Talvacchio, Alberta Derkyi, Jaydira Del Rivero, Geraldine O’Sullivan Coyne, Alice P. Chen, Naris Nilubol, Peter Herscovitch, Frank I. Lin, David Taieb, A. Cahid Civelek, Jorge A. Carrasquillo, Karel Pacak

**Affiliations:** 1 Section On Medical Neuroendocrinology, Eunice Kennedy Shriver National Institute of Child Health and Human Development, National Institutes of Health, Room 1E-3140, CRC, Bldg. 10, 10 Center Dr. MSC-1109, Bethesda, MD 20892-1109 USA; 2 Radiology and Imaging Sciences, Warren Grant Magnuson Clinical Center, National Institutes of Health, Bldg. 10, 10 Center Dr., Bethesda, MD 20892 USA; 3 Nuclear Medicine Division, Radiology and Imaging Sciences, Warren Grant Magnuson Clinical Center, National Institutes of Health, Bldg. 10, 10 Center Dr., Bethesda, MD 20892 USA; 4 Positron Emission Tomography Department, Warren Grant Magnuson Clinical Center, National Institutes of Health, Bldg. 10, 10 Center Dr., Bethesda, MD 20892 USA; 5 Biostatistics and Clinical Epidemiology Service, Warren Grant Magnuson Clinical Center, National Institutes of Health, Bldg. 10, 10 Center Dr., Bethesda, MD 20892 USA; 6 Medical Genetics Branch, National Human Genome Research Institute, National Institutes of Health, Bethesda, MD 20892 USA; 7 Developmental Therapeutics Branch, National Cancer Institute, National Institutes of Health, Room 13C434, Bldg. 10, 10 Center Dr., Bethesda, MD 20892 USA; 8 Division of Cancer Treatment and Diagnosis, National Cancer Institute, National Institutes of Health, Room 8D53, Bldg. 10, 10 Center Dr., Bethesda, MD 20892 USA; 9 Surgical Oncology Program, Center for Cancer Research, National Cancer Institute, Room 4-5952, Bldg. 10, 10 Center Dr., Bethesda, MD 20892 USA; 10 Molecular Imaging Branch, National Cancer Institute, National Institutes of Health, Room 13C442, Bldg. 10, 10 Center Dr., Bethesda, MD 20892 USA; 11 Department of Nuclear Medicine, La Timone University Hospital, CERIMED, Aix-Marseille University, Marseille, France; 12 Nuclear Medicine, Radiology and Radiological Science, Johns Hopkins Medicine, Baltimore, MD USA

**Keywords:** Gallium GA 68 DOTATATE, Fluorodeoxyglucose F18, Neuroendocrine tumors, Pheochromocytoma, Paraganglioma

## Abstract

**Objective:**

To compare the diagnostic performance of [^68^Ga]DOTATATE PET/CT, [^18^F]FDG PET/CT, MRI of the spine, and whole-body CT and MRI for the detection of pheochromocytoma/paraganglioma (PPGL)–related spinal bone metastases.

**Materials and methods:**

Between 2014 and 2020, PPGL participants with spinal bone metastases prospectively underwent [^68^Ga]DOTATATE PET/CT, [^18^F]FDG PET/CT, MRI of the cervical-thoracolumbar spine (MRI_spine_), contrast-enhanced MRI of the neck and thoraco-abdominopelvic regions (MRI_WB_), and contrast-enhanced CT of the neck and thoraco-abdominopelvic regions (CT_WB_). Per-patient and per-lesion detection rates were calculated. Counting of spinal bone metastases was limited to a maximum of one lesion per vertebrae. A composite of all functional and anatomic imaging served as an imaging comparator. The McNemar test compared detection rates between the scans. Two-sided *p* values were reported.

**Results:**

Forty-three consecutive participants (mean age, 41.7 ± 15.7 years; females, 22) with MRI_spine_ were included who also underwent [^68^Ga]DOTATATE PET/CT (*n* = 43), [^18^F]FDG PET/CT (*n* = 43), MRI_WB_ (*n* = 24), and CT_WB_ (*n* = 33). Forty-one of 43 participants were positive for spinal bone metastases, with 382 lesions on the imaging comparator. [^68^Ga]DOTATATE PET/CT demonstrated a per-lesion detection rate of 377/382 (98.7%) which was superior compared to [^18^F]FDG (72.0%, 275/382, *p* < 0.001), MRI_spine_ (80.6%, 308/382, *p* < 0.001), MRI_WB_ (55.3%, 136/246, *p* < 0.001), and CT_WB_ (44.8%, 132/295, *p* < 0.001). The per-patient detection rate of [^68^Ga]DOTATATE PET/CT was 41/41 (100%) which was higher compared to [^18^F]FDG PET/CT (90.2%, 37/41, *p* = 0.13), MRI_spine_ (97.6%, 40/41, *p* = 1.00), MRI_WB_ (95.7%, 22/23, *p* = 1.00), and CT_WB_ (81.8%, 27/33, *p* = 0.03).

**Conclusions:**

[^68^Ga]DOTATATE PET/CT should be the modality of choice in PPGL-related spinal bone metastases due to its superior detection rate.

**Clinical relevance statement:**

In a prospective study of 43 pheochromocytoma/paraganglioma participants with spinal bone metastases, [^68^Ga]DOTATATE PET/CT had a superior per-lesion detection rate of 98.7% (377/382), compared to [^18^F]FDG PET/CT (*p* < 0.001), MRI of the spine (*p* < 0.001), whole-body CT (*p* < 0.001), and whole-body MRI (*p* < 0.001).

**Graphical abstract:**

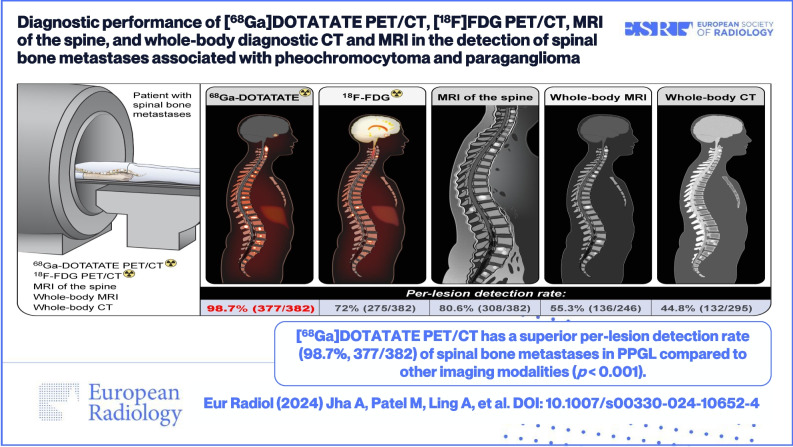

**Key Points:**

*• Data regarding head-to-head comparison between functional and anatomic imaging modalities to detect spinal bone metastases in pheochromocytoma/paraganglioma are limited.*

*•* [^*68*^*Ga*]*DOTATATE PET/CT had a superior per-lesion detection rate of 98.7% in the detection of spinal bone metastases associated with pheochromocytoma/paraganglioma compared to other imaging modalities:* [^*18*^*]F-FDG PET/CT, MRI of the spine, whole-body CT, and whole-body MRI.*

*•* [^*68*^*Ga*]*DOTATATE PET/CT should be the modality of choice in the evaluation of spinal bone metastases associated with pheochromocytoma/paraganglioma.*

## Introduction

Pheochromocytomas and paragangliomas (PPGLs) are rare catecholamine-producing neuroendocrine tumors that cause life-threatening complications [1–3]. Bone metastases are frequently observed in patients with solid tumors and are observed in up to 71% (91/137) of metastatic PPGL patients [4–6]. Further, 20% (26/128) of metastatic PPGL patients have exclusively bony metastases, with the spine being the most common location of bone metastasis (81%, 74/91) [4]. Bone metastases weaken and destroy skeletal tissue and predispose cancer patients to acute and chronic skeletal-related events (SREs) such as bone pain, spinal cord compression, pathological fractures, and/or hypercalcemia [7, 8]. SREs can be the first manifestation of metastatic disease in 31% (15/48) of PPGL patients who develop SREs [4] and spinal cord compression may occur in 20% (5/25) of PPGL patients [9]. The majority of SREs in solid tumors occur within 1 month of diagnosis of bone metastasis [7]. In metastatic PPGL, the median duration between diagnosis of bone metastases and development of the first SRE was 4.3 months, and interval development of a second SRE took a median duration of 9.8 months [4]. SREs not only affect the quality of life, but also increase medical costs, health resource utilization, morbidity, and mortality [7, 10]. Furthermore, vertebral destruction by spinal tumors leads to bony instability and spinal cord compression, with consequent intractable pain, impaired ambulation, neurologic dysfunction, and resultant paralysis [11]. Therefore, prompt diagnosis and intervention are critical in mitigating complications related to bone metastases.

Like other neuroendocrine tumors, PPGLs are known to express somatostatin receptors (SSTR) especially the SSTR2 subtype; DOTATATE is known to demonstrate higher affinity for SSTR2 [12–15]. Currently, ^68^Ga-DOTA(0)-Tyr(3)-octreotate ([^68^Ga]DOTATATE) positron emission tomography/computed tomography (PET/CT) is the imaging modality of choice for the evaluation and the management of patients with metastatic PPGL and demonstrates superior sensitivity over ^18^F-fluoro-2-deoxy-d-glucose ([^18^F]FDG) PET/CT [16]. Alternatively, the recently FDA-approved radiopharmaceutical ^64^Cu-DOTATATE can also be used for PPGL imaging [17]. Furthermore, DOTA peptides, such as DOTATATE and DOTATOC, are not only available for imaging, but also can be labeled with therapeutic beta emitters (Lutetium-177, Yttrium-90) and alpha emitters (Actinium-225, Lead-212) for peptide receptor radionuclide therapy (PRRT) [18–21]. The detection of metastatic PPGLs with [^68^Ga]DOTATATE PET/CT can play a significant role in their medical management, not only because of its diagnostic accuracy, but also to determine patients’ eligibility for PRRT or cold somatostatin analog therapy and to monitor therapeutic response in patients with bone-only disease [13, 18, 22, 23].

Additionally, [^68^Ga]DOTATATE PET/CT was found to be superior to other functional imaging modalities and contrast-enhanced computed tomography (CT) and/or magnetic resonance imaging (MRI) in the detection of patients harboring pathogenic variants in genes encoding succinate dehydrogenase (*SDH)* enzyme subunit B (*SDHB*)–related, subunit A (*SDHA*)–related, pediatric *SDHx*–related, and apparently sporadic metastatic PPGL including bone metastases [24–27]. However, thus far, head-to-head comparison between these radiopharmaceuticals and MRI of the spine to detect spinal bone metastases has not been performed. Accordingly, this study aims to evaluate and compare the diagnostic performance of [^68^Ga]DOTATATE PET/CT to [^18^F]FDG PET/CT, MRI of the cervical-thoracolumbar spine (MRI_spine_), whole-body diagnostic MRI of the neck and thoraco-abdominopelvic regions (MRI_WB_), and whole-body diagnostic CT of the neck and thoraco-abdominopelvic regions (CT_WB_) for the detection of spinal bone metastases in PPGL.

## Materials and methods

The study protocol was approved by the Institutional Review Board of the *Eunice Kennedy Shriver* National Institute of Child Health and Human Development (ClinicalTrials.gov Identifier: NCT00004847). Participants were prospectively enrolled between January 2014 and March 2020. Written informed consent from adult participants, or parents along with informed assent from pediatric participants, was obtained for all clinical, genetic, biochemical, and imaging studies. Our institution complies with all applicable laws, regulations, and policies concerning privacy and confidentiality.

### Eligibility criteria

The inclusion criteria were (1) age ≥ 10 years, (2) confirmed histopathologic diagnosis of PPGL with presence/suspicion of spinal bone metastases, (3) MRI_spine_ and functional imaging with [^68^Ga]DOTATATE PET/CT and [^18^F]FDG PET/CT, all performed at our institution and within 6 months of each other, and (4) whole-body imaging with either CT_WB_ or MRI_WB_. Participants were excluded if pregnant or breastfeeding, or found to have a tumor type other than PPGL.

### Study design

This was a prospective, open-label single-center study.

### Imaging studies and techniques

[^68^Ga]DOTATATE was manufactured under an investigational new drug application. PET/CT scans from the upper thighs to the skull were performed 60.4 ± 2.3 min after intravenous injection of a mean administered activity of 190.2 ± 18.0 MBq of [^68^Ga]DOTATATE and 59.5 ± 2.1 min after 280.1 ± 38.8 MBq of [^18^F]FDG. For [^18^F]FDG, participants fasted at least 4 h before injection, and their mean blood glucose was 101.4 ± 29.6 mg/dL. All PET/CT scans were performed on Biograph-mCT (Siemens Medical Solutions) 64 or 128 PET/CT scanners. [^68^Ga]DOTATATE PET/CT images were reconstructed on a 400 × 400 image matrix with 1.5-mm slice thickness and [^18^F]FDG PET/CT images were reconstructed on a 256 × 256 with 3-mm thickness, using an iterative reconstruction algorithm provided by the manufacturer, utilizing time of flight. Low-dose CT without oral or intravenous contrast was performed for attenuation correction and anatomic co-registration.

MRI_spine_ of the cervical-thoracolumbar spine was performed using a 3.0**-**Tesla whole-body MRI scanner (Achieva, Philips Healthcare). The acquisition protocol consisted of sagittal T1-weighted, sagittal short tau inversion time inversion recovery (STIR) images, and axial T2-weighted images of the spine, and contrast was not administered. Slice thickness was 3 mm for all studies.

MRI_WB_ of the neck and thoraco-abdominopelvic regions were obtained with 1.5- and 3-Tesla scanners (Achieva, Philips Medical Systems; Aera 1.5 Tesla or Siemens Verio 3 Tesla, Siemens Medical Solutions). Imaging protocols varied by body part. The neck included axial STIR and/or T2-weighted images as well as axial T1-weighted or Dixon and sagittal Dixon pre- and post-contrast images. Chest studies included axial T2-weighted, STIR or fat-saturated T2 images, axial fat-saturated T1 pre-contrast and multiphase post-contrast, and coronal fat-saturated T1 post-contrast images. Coronal pre-contrast T2, axial diffusion-weighted imaging (DWI), and non-breath-hold axial T2* were each sometimes included. Abdominal studies also included axial DWI, axial T2-weighted images with and without fat saturation, axial T1 in and out of phase, and multiphase, multiplanar T1-weighted images before and after contrast injection. Pelvic studies included axial T2-weighted or STIR, axial DWI and T1-weighted, and sagittal fat-saturated T2-weighted images prior to and multiplanar fat-saturated T1-weighted images following contrast injection. Slice thickness was no greater than 5 mm for all neck studies, and no greater than 6 mm for all thoraco-abdominopelvic scans. Post-gadolinium contrast images as thin as 1 mm in the neck and 3 mm for other body parts were obtained for most scans. Pelvic imaging was typically performed contemporaneously with abdominal imaging, sharing a single contrast injection. Similarly, chest and neck imaging were typically performed contemporaneously, and shared a single contrast injection. Each contrast injection consisted of 0.1 mmol/kg of a gadolinium-based contrast agent.

CT_WB_ of the neck and thoraco-abdominopelvic regions were all performed using Siemens Somatom Force or Siemens Definition Flash (Siemens Medical Solutions) or Toshiba Aquilion One scanners (Canon Medical Systems). Slice thickness was 2 mm for all studies. All studies were performed with rapid intravenous infusion (injection rate 2 mL/s) of a nonionic low osmolality water-soluble contrast agent (Isovue 300, Bracco Diagnostics).

### Analysis of data

All PET/CT studies were interpreted by an experienced nuclear medicine physician (J.A.C., 36 years of experience), all whole-body diagnostic CT_WB_ and MRI_WB_ studies were interpreted by an experienced radiologist (A.L., 36 years of experience), and all MRI_spine_ were interpreted by an experienced neuroradiologist (R.S., 21 years of experience). Moreover, they had 22, 16, and 5 years of experience, respectively, in interpreting PPGL imaging and were blinded to all other imaging and clinical data except for the suspected diagnosis, sex, and age of the patient. [^68^Ga]DOTATATE and [^18^F]FDG PET/CT images were all reviewed using MIM software (version 7.0.7). Orthogonal views as well as maximum intensity projection (MIP) images were reviewed from each modality. Typically, [^68^Ga]DOTATATE PET/CT images of the spine were reviewed first followed by review of [^18^F]FDG PET/CT.

### Standard of reference

Histologic proof of all spinal bone metastases was not feasible. Therefore, a composite of all the imaging studies served as an imaging comparator or reference standard for the calculation of detection rates [24, 25, 27]. A “positive” result on both functional PET/CT imaging ([^68^Ga]DOTATATE and [^18^F]FDG) or at least on one functional PET/CT ([^68^Ga]DOTATATE or [^18^F]FDG) imaging and on one of the anatomic imaging modalities (MRI_spine_ or MRI_WB_ or CT_WB_) was considered as true disease. A positive lesion found only on one imaging modality while negative on all other imaging modalities was considered a false-positive imaging result.

### Statistical analysis

Results are given as means with 95% confidence intervals (CIs) unless stated otherwise. Per-patient and per-lesion detection rates of [^68^Ga]DOTATATE PET/CT, [^18^F]FDG PET/CT, MRI_spine_, MRI_WB_, and CT_WB_ were calculated. A participant was considered abnormal or “positive” regardless of the number of positive lesions present; counting of spinal bone metastases was limited to a maximum of one lesion per vertebrae. Cochran’s *Q* test was used to perform a global comparison of detection rates across the imaging modalities. Since MRI_WB_ and CT_WB_ were not performed in all participants, Cochran’s *Q* test was performed with and without MRI_WB_ and CT_WB_. The McNemar test was used to compare detection rates between [^68^Ga]DOTATATE PET/CT and the other imaging modalities. Two-sided *p* values were reported. Statistical analyses (A.J. and N.S.) were performed using SPSS v29.0 (IBM Corp.), SAS v9.4 (SAS Institute), and StatXact (Cytel Inc.).

## Results

**Fig. 1:**
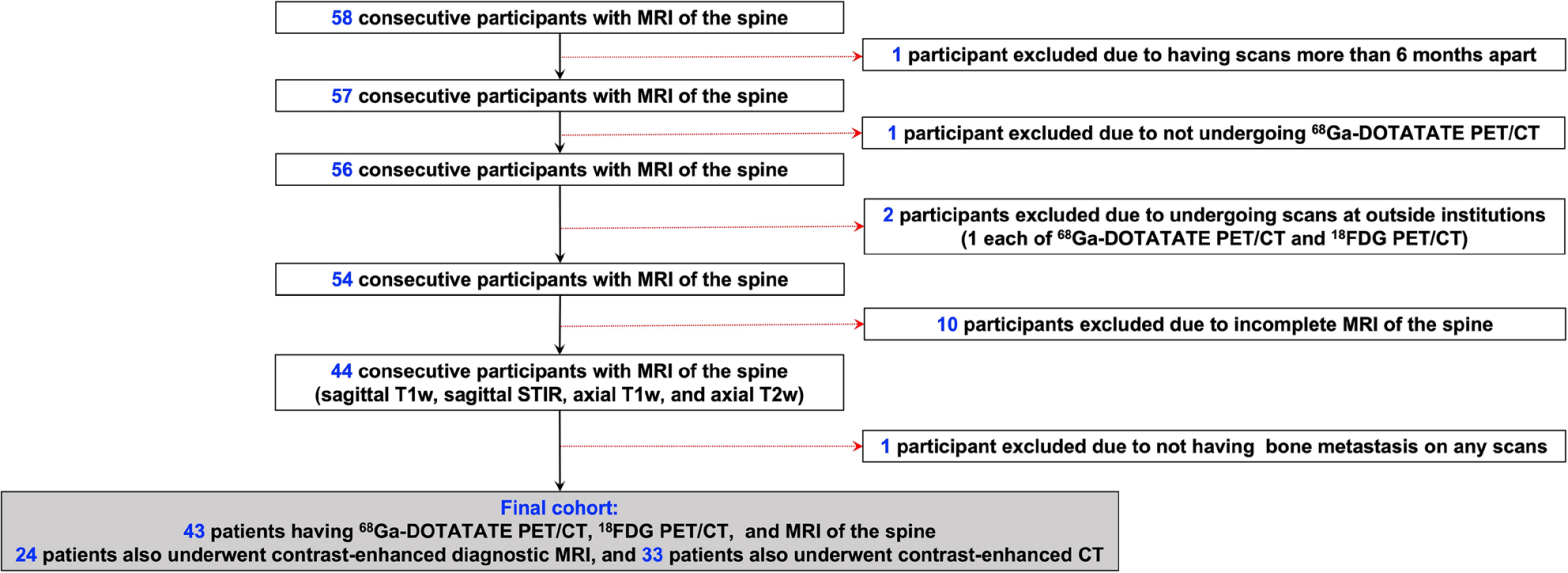
Schematic diagram representing enrollment of participants in the study. The diagram of flow of participants’ inclusion and exclusion

Of the 58 consecutive participants undergoing MRI_spine_ for the evaluation of spinal bone metastases, 15 participants were excluded (Fig. 1). One participant each was excluded for having scans more than 6 months apart and not undergoing [^68^Ga]DOTATATE PET/CT. Two of 15 participants underwent scans (one participant each with [^68^Ga]DOTATATE and [^18^F]FDG PET/CT) at outside institutions and therefore were excluded. Ten of 16 participants were excluded due to incomplete MRI_spine_ (one did not complete the scan due to bone pain; T1-weighted and STIR images were not obtained in one and eight participants, respectively). Finally, one participant did not have any biochemical or imaging evidence of PPGL. Further, eight vertebral bodies in three participants were excluded from analysis due to the presence of artifacts on anatomic imaging caused by spinal hardware. One participant did not undergo either MRI_WB_ or CT_WB_ as they had an outside CT_WB_.

A total of 43 participants (median (IQR) age, 40 (28–54) years; females, 22) with MRI_spine_ were included in the study who also underwent [^68^Ga]DOTATATE PET/CT and [^18^F]FDG PET/CT. Thirty-three (76.7%) participants also underwent CT_WB_, and 24 (55.8%) underwent MRI_WB_. The median (IQR) duration between [^68^Ga]DOTATATE PET/CT and [^18^F]FDG PET/CT was 4 (2–6) days, between [^68^Ga]DOTATATE PET/CT and MRI_spine_ 5 (1–35) days, between [^68^Ga]DOTATATE PET/CT and MRI_WB_ 4 (2–5) days, and between [^68^Ga]DOTATATE PET/CT and CT_WB_ 2 (1–7) days. SREs were reported in 29 (67.4%) participants. Thirty-two (74.4%) participants harbored germline pathogenic variants in PPGL susceptibility genes and 11 (25.6%) participants tested negative for germline mutations in PPGL susceptibility genes. Thirty-four (79%) participants had biochemical elevations in plasma catecholamines or their metabolites. Nineteen (44.2%) participants had an adrenergic phenotype (biochemical elevation in epinephrine or its metabolite, metanephrine), 28 (65.1%) had a noradrenergic phenotype (biochemical elevation in norepinephrine or its metabolite, normetanephrine), and 20 (46.5%) had a dopaminergic phenotype (biochemical elevation in dopamine or its metabolite, methoxytyramine). Thirty (69.8%) participants also had elevated plasma chromogranin A and five participants did not have biochemical elevations in plasma catecholamines or their metabolites. The demographic and clinical data is summarized in Table 1.

**Table 1:** Summary of participant characteristics

Characteristics	Value
No. of participants	43 (100)
No. of females	22 (51.2)
Age at diagnosis (y)*	22 (15–43)
Age at enrollment (y)*	40 (28–54)
Location of primary tumor	
Pheochromocytoma	11 (25.6)
Paraganglioma	29 (67.4)
Both pheochromocytoma and paraganglioma	3 (7.0)
Skeletal-related events related to spine before enrollment	29 (67.4)
Pain in vertebrae	16 (37.2)
Compression or pathologic fracture	5 (11.6)
Cord compression or spinal stenosis	6 (14.0)
Surgery and/or stabilization of vertebra/e	7 (16.3)
Radiation therapy to spinal vertebra/e	10 (23.3)
Genetic testing results	
*SDHB*	21 (48.8)
*SDHA*	4 (9.3)
*SDHD*	3 (7.0)
*SDHC*	1 (2.3)
*HIF2A*	1 (2.3)
*NF1*	1 (2.3)
* FH*	1 (2.3)
Negative for pheochromocytoma susceptibility genes	11 (25.6)
Biochemical elevations	34 (79.1)
Epinephrine/metanephrine	19 (44.2)
Norepinephrine/normetanephrine	28 (65.1)
Dopamine/methoxytyramine	20 (46.5)
Chromogranin A	30 (69.8)
Treatment received	41 (95.3)
Surgery	38 (88.4)
Chemotherapy (CVD, temozolomide, CAPTEM, TKIs)	13 (30.2)
Somatostatin analogs	6 (14.0)
Targeted radiotherapy (^131^I-MIBG, PRRT)	13 (30.2)
External radiation therapy	14 (32.6)
Immunotherapy	1 (2.3)
Bisphosphonates/denosumab	10 (27.9)

Unless otherwise indicated, data are numbers of participants and data in parentheses are percentages

*Y* years, *SDHA-D* germline pathogenic variants in succinate dehydrogenase subunits A-B, *HIF2A* hypoxia-inducible factor-2α, *NF1* neurofibromatosis type 1, *FH* fumarate hydratase, *CVD* cyclophosphamide, vincristine, and dacarbazine, *CAPTEM* capecitabine and temozolomide, *TKIs* tyrosine kinase inhibitors, *PRRT* peptide receptor radionuclide radiotherapy, ^*131*^*I-MIBG* ^131^I-metaiodobenzylguanidine

*Data expressed as medians with interquartile range in parentheses

**Fig. 2:**
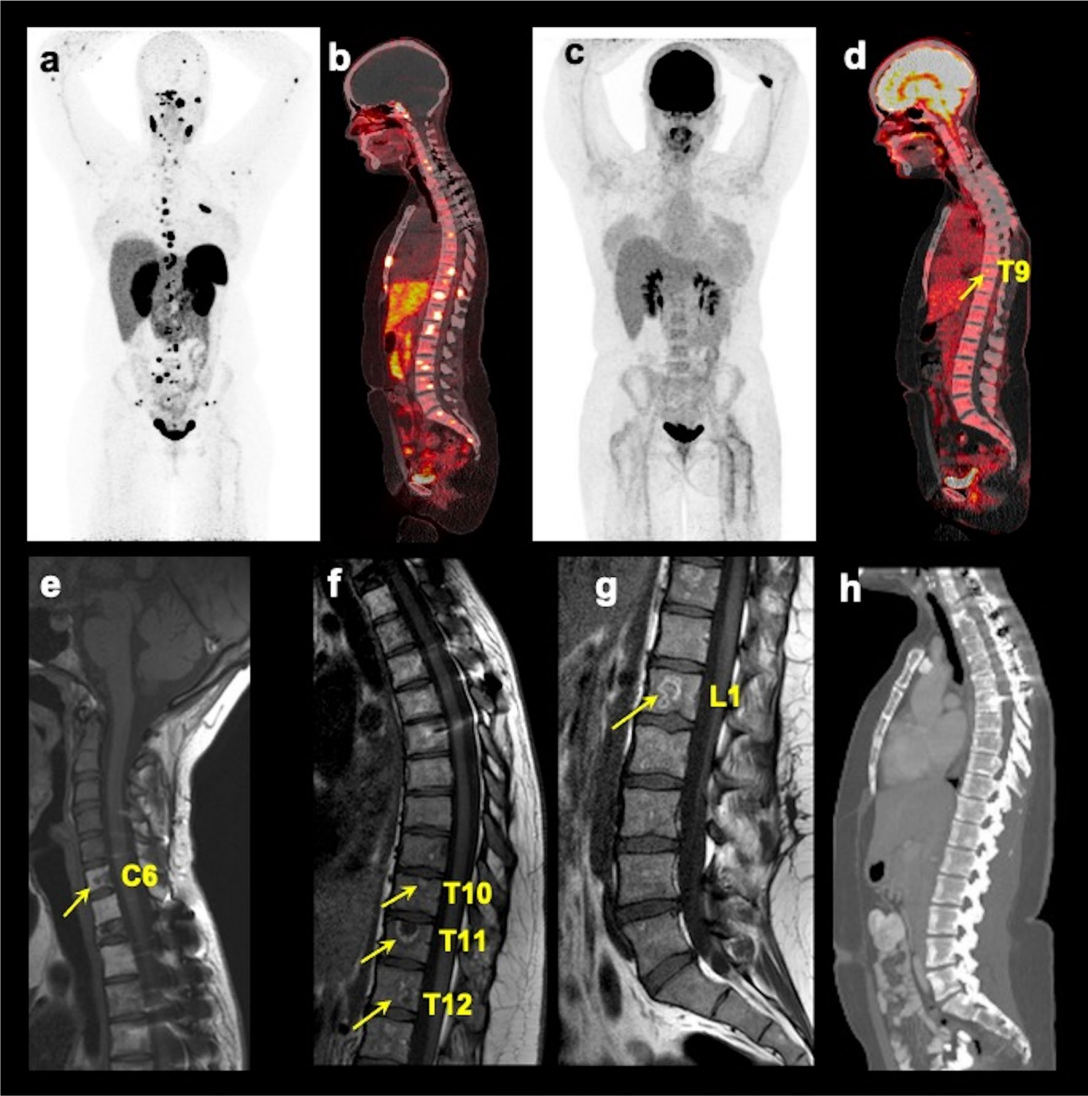
Multimodality imaging of spinal bone metastases in a pheochromocytoma/paraganglioma participant. The images of whole-body ^68^Ga-DOTA(0)-Tyr(3)-octreotate ([^68^Ga]DOTATATE; **a** anterior maximum intensity projection (MIP) and (**b**) fused sagittal PET/CT), ^18^F-fluoro-2-deoxy-d-glucose ([^18^F]FDG; **c** MIP and (**d**) fused sagittal PET/CT), T1-weighted sagittal MRI of the cervical (**e**), thoracic (**f**), and lumbar (**g**) spine, and contrast-enhanced CT (**h** sagittal) of a 25-year-old woman with germline pathogenic variant in a gene encoding for succinate dehydrogenase B subunit are shown. This figure shows superiority of [^68^Ga]DOTATATE PET/CT in the detection of additional spinal bone metastases at C2, C5, C7, T1-2, T4-8, L2, and L5 compared to [^18^F]FDG PET/CT in detecting spinal bone metastases at T9 (arrow on fused sagittal image and not appreciated on MIP image) and L4 (not appreciated on MIP and fused sagittal images), and MRI spine at C6, T10-12, and L1 (arrows), respectively. The whole-body CT was read negative for any spinal bone metastases. This participant did not undergo whole-body MRI

Of the 43 participants, 41 (95.3%) were positive for spinal bone metastases, with 382 lesions on the imaging comparator. The remaining two participants were positive only on [^68^Ga]DOTATATE PET/CT and not found positive on either [^18^F]FDG PET/CT or any other anatomic imaging, and therefore were classified as false positive. [^68^Ga]DOTATATE PET/CT demonstrated a per-lesion detection rate of 377/382 (98.7%, 95% confidence interval (CI) 97.0–99.6%) which was different compared to other imaging modalities at the global level (*p* < 0.001 for all with and without MRI_WB_ and CT_WB_). Compared to [^68^Ga]DOTATATE PET/CT, [^18^F]FDG PET/CT, MRI_spine_, MRI_WB_, and CT_WB_ showed lower per-lesion detection rates of 275/382 (72.0%, 95% CI 67.2–76.4%; *p* < 0.001), 308/382 (80.6%, 95% CI 76.3–84.5%; *p* < 0.001), 136/246 (55.3%, 95% CI 48.8–61.6%; *p* < 0.001), and 132/295 (44.8%, 95% CI 39.0–50.6%; *p* < 0.001), respectively. The number of lesions that were classified as false positive by [^68^Ga]DOTATATE PET/CT, [^18^F]FDG PET/CT, MRI_spine_, MRI_WB_, and CT_WB_ was 81, 0, 75, 17, and 16, respectively. The per-patient detection rate of [^68^Ga]DOTATATE PET/CT was 41/41 (100%, 95% CI 91.4–100%). On global comparison, [^68^Ga]DOTATATE PET/CT was better than other modalities when compared without MRI_WB_ and CT_WB_ (*p* = 0.02) but not different when MRI_WB_ and CT_WB_ were included (*p* = 0.41). [^18^F]FDG PET/CT, MRI_spine_, and MRI_WB_ showed per-patient detection rates of 37/41 (90.2%, 95% CI 76.9–97.3%; *p* = 0.13), 40/41 (97.6%, 95% CI 87.1–99.9%; *p* = 1.00), and 22/23 (95.7%, 95% CI 78.1–99.9%; *p* = 1.00), respectively, that were lower but not different from [^68^Ga]DOTATATE PET/CT. However, CT_WB_ had a lower detection rate of 27/33 (81.8%, 95% CI 64.5–93.0%; *p* = 0.03) (Table 2). Further, [^68^Ga]DOTATATE PET/CT was found to detect greater (26/41; 63.4%, 95% CI 46.9–77.9%) or equal (14/41; 34.2%, 95% CI 20.1–50.6%) true-positive lesions compared to [^18^F]FDG PET/CT in 40/41 (97.6%, 95% CI 87.1–99.9%) participants, whereas [^68^Ga]DOTATATE PET/CT was found to detect greater (27/41; 65.9%, 95% CI 49.4–79.9%) or equal (14/41; 34.2%, 95% CI 20.1–50.6%) true-positive lesions compared to MRI_spine_ in 41/41 (100.0%, 95% CI 91.4–100.0%) participants (Table 3). Additionally, [^68^Ga]DOTATATE PET/CT was found to detect greater (21/23; 91.3%, 95% CI 72.0–98.9%) or equal (2/23; 8.7%, 95% CI 1.1–28.0%) true-positive lesions compared to MRI_WB_ in 23/23 (100.0%, 95% CI 85.2–100.0%) participants, whereas [^68^Ga]DOTATATE PET/CT was found to detect greater (28/33; 84.8%, 95% CI 68.1–94.9%) or equal (5/33; 15.2%, 95% CI 5.1–31.9%) true-positive lesions compared to CT_WB_ in 33/33 (100.0%, 89.4–100.0%) participants (Table 3). Lastly, [^68^Ga]DOTATATE PET/CT detected a median (range) of 2 (1–11) lesions more than MRI_spine_ in 26 participants where [^68^Ga]DOTATATE PET/CT was superior to MRI_spine_. A representative figure is shown demonstrating the superior performance of [^68^Ga]DOTATATE PET/CT compared to various imaging modalities in a participant (Fig. 2).

**Table 2:** Per-lesion and per-patient detection rate (%) of spinal bone metastases utilizing [^68^Ga]DOTATATE PET/CT, [^18^F]FDG PET/CT, MRI of the spine, whole-body MRI, and whole-body CT in pheochromocytoma/paraganglioma participants

Detection rates	[^68^ Ga]DOTATATE PET/CT	[^18^F]FDG PET/CT	MRI of the spine	Whole-body MRI	Whole-body CT
Per-lesion	377/382 (98.7%, 97.0–99.6%)	275/382 (72.0%, 67.2–76.4%)	308/382 (80.6%, 76.3–84.5%)	136/246 (55.3%, 48.8–61.6%)	132/295 (44.8%, 39.0–50.6%)
Per-patient	41/41 (100%, -91.4–100%)	37/41 (90.2%, 76.9–97.3%)	40/41 (97.6%, 87.1–99.9%)	22/23 (95.7%, 78.1–99.9%)	27/33 (81.8%, 64.5–93.0%)

Values in the table are detection rates expressed in ratios, defined as the number of lesions or participants detected by the imaging modality compared to the total number of lesions or participants evaluated by that modality. In parentheses are the percentages along with 95% confidence intervals

**Table 3:** Relative performance of [^68^Ga]DOTATATE PET/CT compared to other imaging modalities in detecting number of lesions per patient

No. of participants	[^68^ Ga]DOTATATE PET/CT			
	**Greater**	**Equal**	**Lesser**	**Greater or equal**
[^**18**^**F]FDG PET/CT**	26/41 (63.4%, 46.9–77.9%)	14/41 (34.2%, 20.1–50.6)	1/41 (2.4%, 0.1–12.9%)	40/41 (97.6%, 87.1–99.9%)
**MRI of the spine**	27/41 (65.9%, 49.4–79.9%)	14/41 (34.2%, 20.1–50.6)	0/41 (0.0%, 0.0–8.6%)	41/41 (100%, 91.4–100.0%)
**Whole-body MRI**	21/23 (91.3%, 72.0–98.9%)	2/23 (8.7%, 1.1–28.0%)	0/23 (0.0%, 0.0–14.8%)	23/23 (100%, 85.2–100.0%)
**Whole-body CT**	28/33 (84.8%, 68.1–94.9%)	5/33 (15.2%, 5.1–31.9%)	0/33 (0.0%, 0.0–10.6%)	33/33 (100%, 89.4–100.0%)

Values in the table are expressed in ratios, defined as the number of participants of [^68^Ga]DOTATATE PET/CT detecting greater, equal, lesser, and greater or equal number of lesions per participant compared to [^18^F]FDG PET/CT, MRI of the spine, contrast-enhanced whole-body MRI, and contrast-enhanced CT. In parentheses are the percentages along with 95% confidence intervals

## Discussion

In this prospective study, we performed an intraindividual comparison of spinal bone metastases using [^68^Ga]DOTATATE PET/CT, [^18^F]FDG PET/CT, MRI_spine_, MRI_WB_, and CT_WB_ in 43 participants. A composite of both anatomic and functional imaging served as an imaging comparator consistent with previous studies [24, 25, 27]. Patients with bone metastases experience SREs soon after diagnosis, which are associated with poor quality of life [4, 7, 10, 11]. Therefore, prompt diagnosis and intervention are critical to reduce morbidity/complications. [^68^Ga]DOTATATE PET/CT had a superior per-lesion detection rate of 98.7% (377/382), which was substantially higher compared to MRI_spine_ (80.6%, *p* < 0.001), [^18^F]FDG PET/CT (72.0%, *p* < 0.001), MRI_WB_ (55.3%, *p* < 0.001), and CT_WB_ (44.8%, *p* < 0.001). However, there was no difference in per-patient detection rate between [^68^Ga]DOTATATE PET/CT and other imaging modalities except CT_WB_. Furthermore, [^68^Ga]DOTATATE PET/CT detected a greater or equal number of true-positive lesions compared to all imaging modalities in all participants except one in whom [^18^F]FDG PET/CT detected one more lesion than [^68^Ga]DOTATATE PET/CT.

**Fig. 3:**
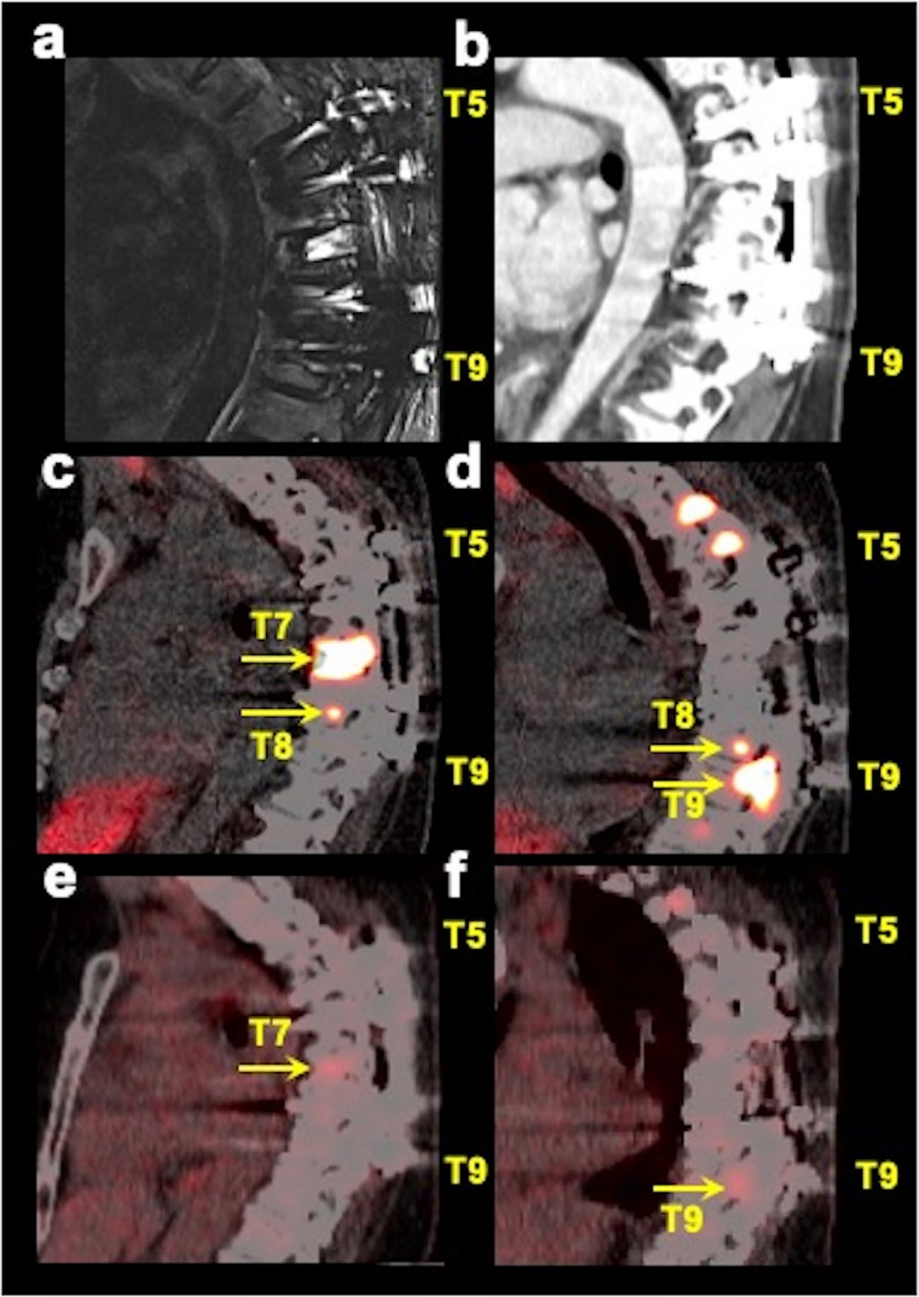
Multimodality imaging in a spinal bone metastatic pheochromocytoma/paraganglioma participant with spinal hardware. The images of sagittal short tau inversion recovery (STIR, **a**) MRI, contrast-enhanced CT (**b** sagittal), fused sagittal ^68^Ga-DOTA(0)-Tyr(3)-octreotate ([^68^Ga]DOTATATE; **c**, **d** images at two different sagittal planes) PET/CT, and fused sagittal ^18^F-fluoro-2-deoxy-d-glucose ([^18^F]FDG PET/CT; **e**, **f** images at the same two sagittal planes as [^68^Ga]DOTATATE PET/CT) focused on thoracic spine of a 48-year-old woman with negative germline testing in pheochromocytoma and paraganglioma susceptibility genes are shown. This participant had spinal hardware placed at the T5–T9 vertebrae which therefore were excluded from the analysis due to the associated artifacts on MRI and CT. However, PET/CT imaging clearly is advantageous in the evaluation of patients with spinal bone hardware as demonstrated by the spinal bone metastases at T7–T9 on [^68^Ga]DOTATATE PET/CT (arrows) and at T7 and T9 on [^18^F]FDG PET/CT (arrows), where spinal hardware is placed. The uptake on [^18^F]FDG PET/CT is comparatively much fainter compared to [^68^Ga]DOTATATE PET/CT. This participant did not undergo whole-body MRI

**Fig. 4:**
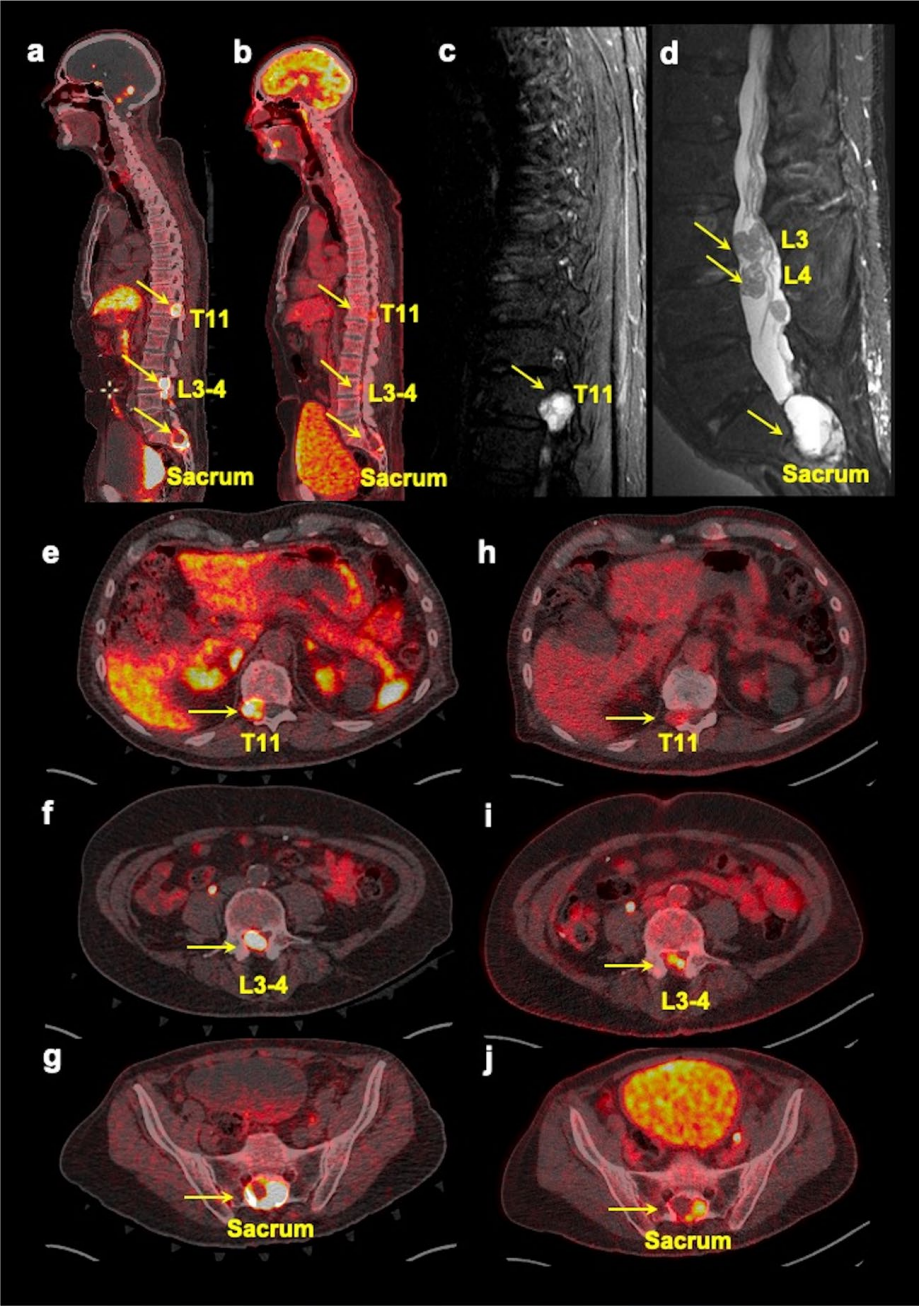
Multimodality imaging in a spinal bone metastatic pheochromocytoma/paraganglioma participant with spinal canal involvement. The whole-body fused sagittal (**a**, **b**) images of ^68^Ga-DOTA(0)-Tyr(3)-octreotate ([^68^Ga]DOTATATE, **a**) PET/CT, ^18^F-fluoro-2-deoxy-d-glucose ([^18^F]FDG, **b**) PET/CT, and sagittal short tau inversion recovery (STIR, **c**, **d**) images of thoracic (**c**), lumbar (**d**), and axial (**e**–**j**) fused PET/CT images of [^68^Ga]DOTATATE (**e**–**g**) and [^18^F]FDG (**h**–**j**) of a 71-year-old man with negative germline testing in pheochromocytoma and paraganglioma susceptibility genes. This figure shows spinal bone metastases (arrows, **a**–**j**) in T11, L3–L4, and sacrum with spinal canal involvement. In such cases, a dedicated MRI of the spine (showing intradural tumors, here) to derive more anatomic information should be obtained

Our results are consistent with the reported per-lesion sensitivities of [^68^Ga]DOTATATE PET/CT in the detection of bone metastases in *SDHB-*related (95/98, 96.9%, prospective evaluation), *SDHA-*related (208/223, 93.3%, prospective evaluation), pediatric *SDHx* (62/64, 96.9%, retrospective evaluation), and apparently sporadic-related (199/199, 100%, prospective evaluation) PPGLs, which were found to be higher compared to [^18^F]FDG PET/CT (92.9% (91/98), 84.3% (188/223), 75% (48/64), and 50.3% (100/199), respectively), and whole-body CT and/or MRI (83.7% (82/98), 51.9% (110/212), 68.8% (44/64), and 79.4% (158/199), respectively) [24–27]. These studies reported combined findings on CT_WB_ or MRI_WB_ but did not include MRI_spine_. In another prospective evaluation in 25 PPGL participants, ^68^Ga-DOTATOC (another DOTA peptide) PET/MRI detected all 28 bone metastases compared to 23 by ^68^Ga-DOTATOC PET/CT [28]. The participants in that study underwent same-day consecutive acquisition of PET/CT and PET/MRI scans, respectively, following a single ^68^Ga-DOTATOC injection. These results suggest that PET/MRI is superior to PET/CT for the evaluation of spinal bone metastases, especially in pediatric patients in whom limiting radiation is a concern given their lifelong imaging requirement. Additionally, PET/CT imaging may aid in detecting metastases in vertebrae where spinal hardware has been placed which may not be detected on anatomic imaging due to associated artifacts (Fig. 3). If more anatomic information is required for surgical/radiation therapy planning or cord compression or spinal stenosis is suspected, then dedicated MRI_spine_ of that region should be obtained (Fig. 4).

To note, DWI and contrast-enhanced imaging were not part of the MRI_spine_ protocol. MRI_spine_ in our institution is a clinically indicated study and does not include these sequences for detection of spinal bone metastases. Moreover, these sequences were included for the abdominopelvic region of MRI_WB_ and did not improve the detection of spinal bone metastases.

There are some limitations of this study. First, the study cohort is modest in size although relatively large considering the rarity of spinal bone metastatic PPGLs. Second, there may be selection bias as only participants with known or suspicious spinal metastases underwent accrual, thereby excluding asymptomatic patients with spinal metastases. However, it was not feasible to perform MRI_spine_ on all the participants getting enrolled in our protocol. Lastly, even though the chosen imaging comparator likely provides a close approximation of “truth,” false-positive and false-negative findings could not be excluded [27]. Histological proof was neither feasible nor ethical for the confirmation of metastatic lesions [29].

## Conclusions and future directions

[^68^Ga]DOTATATE PET/CT demonstrated superiority in the detection of spinal bone metastases compared to [^18^F]FDG PET/CT, MRI_spine_, MRI_WB_, and CT_WB_ indicating that it should be the imaging modality of choice when looking for metastatic spine disease associated with PPGL. Not only is it more sensitive than the other modalities, but it is also useful in the detection of primaries, soft tissue metastases, and the treatment planning and response assessment of targeted radionuclide therapy (Radium-223 dichloride, Lutetitium-177/Yttrium-90/Actinium-225/Lead-212-DOTA-analogs) in patients with bone-only metastatic PPGL in whom response evaluation using anatomic imaging is challenging. Future studies may include diffusion-weighted and post-contrast images in the MRI_spine_ protocol and determine its performance against [^68^Ga]DOTATATE PET/CT.

## Abbreviations

[^18^F]FDG: ^18^F-Fluoro-2-deoxy-d-glucose
[^68^Ga]DOTATATE: ^68^Ga-DOTA(0)-Tyr(3)-octreotate
CT_WB_: Whole-body diagnostic CT of the neck and thoraco-abdominopelvic regions
FH: Fumarate hydratase
HIF2A: Hypoxia-inducible factor-2α
MRI_spine_: MRI of the cervical-thoracolumbar spine
MRI_WB_: Whole-body diagnostic MRI of the neck and thoraco-abdominopelvic regions
NF1: Neurofibromatosis type 1
PPGLs: Pheochromocytomas and paragangliomas
SDHA-D: Succinate dehydrogenase subunits A-D
SREs: Skeletal-related events

## Declarations

### Guarantor

The scientific guarantor of this publication is Dr. Karel Pacak.

### Statistics and biometry

One of the authors, Ninet Sinaii is a biostatistician, has significant statistical expertise.

### Study subjects or cohorts overlap

No.

### Methodology

• prospective

• diagnostic or prognostic study

• performed at one institution
